# A niched Pareto genetic algorithm for finding variable length regulatory motifs in DNA sequences

**DOI:** 10.1007/s13205-011-0040-6

**Published:** 2011-12-09

**Authors:** Shripal Vijayvargiya, Pratyoosh Shukla

**Affiliations:** 1 Department of Computer Science and Engineering, Birla Institute of Technology, Mesra Extension Center Jaipur, 27, Malviya Industrial Area, Jaipur, 302017 Rajasthan India; 2 Department of Biotechnology, Birla Institute of Technology, Mesra (Ranchi), 835215 Jharkhand India

**Keywords:** Motif, TFBS, Binding sites, Multi-objective and genetic algorithm

## Abstract

The transcription factor binding sites also called as motifs are short, recurring patterns in DNA sequences that are presumed to have a biological function. Identification of the motifs from the promoter region of the genes is an important and unsolved problem specifically in the eukaryotic genomes. In this paper, we present a niched Pareto genetic algorithm to identify the regulatory motifs. This approach is based on the maximization of two objectives of the problem that is the motif length and the consensus similarity score. A long motif means it is less likely to be a false motif. The similarity score represents a motifs probability of conservation in a given set of sequences. Proposed method can find multiple, variable length motifs. In this method, we represented a candidate motif as a combination of length and starting position of the motif in each sequence of the co-regulated genes. This enables the algorithm to identify multiple motifs of variable length. We applied this approach on various data sets and the results show that it can find multiple motifs of variable length in co-regulated genes.

## Introduction

Understanding the regulatory networks of higher organisms is one of the main challenges of functional genomics. Gene regulation is a finely controlled mechanism. The main part of regulation is performed by the specific proteins called transcription factors (TFs) binding to a specific transcription factor binding sites (TFBS), in regulatory regions associated with genes. A TFBS is also known as motif. A motif is a pattern of nucleotide bases or amino acids, which captures a biologically meaningful feature common to a group of nucleic acid or protein sequences. Regulatory motifs capture the patterns of DNA bases responsible for controlling when and where a gene is expressed. Typically, regulatory motifs describe TFBSs embedded in the DNA sequences upstream of a gene’s transcription start site (TSS). More rarely, regulatory signals may occur downstream of the TSS and even within coding sequences. Many well-characterized motifs, such as the TATA box, occur proximal to the TSS (Lones and Tyrrell 2007).

Identification of the regulatory regions and binding sites is a prerequisite for understanding gene regulation (Lockhart and Winzeler 2000). Initially, the experimental techniques like DNAse footprinting assay and the Electrophoretic Mobility Shift Assay (EMSA) have been used to discover and analyze DNA binding sites. However, the development of DNA microarrays and fast sequencing techniques has led to new methods for in vivo identification of binding sites, such as ChIP-chip and ChIP-Seq (Elnitski et al. 2006). Experimental identification and verification of such elements is challenging and costly; therefore, much effort has been put into the development of computational approaches. A good computational method can potentially provide high-quality prediction of the binding sites and reduce the time required for experimental verification.

Computational discovery of the regulatory elements is possible because they occur several times in the same genome, and they may be evolutionary conserved (Sandve and Drabløs 2006). This means that searching for overrepresented motifs across regulatory regions may discover novel regulatory elements. However, this simple looking problem turns out to be a tough problem, made difficult by a low signal-to-noise ratio. This is because of the poor conservation and short length of the transcription factor binding sites in comparison with the length of promoter sequences. Recent reviews have noted some important limitations of existing tools for regulatory motif discovery like, the limited applicability of current nucleotide background models, rapid failure with increasing sequence length and a tendency to report false positives rather than true transcription factor binding sites (Tompa et al. 2005; Hu et al. 2005).

Motifs or TFBSs are generally represented as the consensus IUPAC strings, position frequency matrices (PFMs), position weight matrices (PWMs) or position-specific scoring matrices (PSSMs) in the databases. The motif data are modeled as PFM by aligning identified sites and counting the frequency of each base pair at each position of the alignment. Moreover, by using sequence logos, PWM can be displayed with color and height proportional to the base pair frequency and information content for each position by formulas. Known regulatory motif profiles are cataloged in databases such as TRANSFAC (Matys et al. 2003) and JASPAR (Sandelin et al. 2004).

We used a niched Pareto genetic algorithm for regulatory motif discovery. The algorithm uses multi-objective representation of a motif that enables the algorithm to find out Pareto-optimal solution set of variable length motifs. “Existing methods” section contains a brief survey of various techniques and algorithms used to solve the motif finding problem. “Materials and methods” section explains the method and it’s components like representation & initialization, selection, crossover, mutations, fitness objectives and score function. Next section contains the simulation results followed by conclusion.

## Existing methods

Identification of regulatory motifs in upstream region of co-regulated genes or orthologous genes is a challenging problem of computational biology. In the last few years, many algorithms were proposed to find solutions for motif discovery. According to a survey (Das and Dai 2007), two major strategies exist to discover repeating sequence patterns occurring in both DNA and protein sequences: enumeration and probabilistic sequence modeling. Enumeration strategies rely on word counting to find words that are overrepresented. Probabilistic model-based methods represent the pattern as a matrix, called a motif, consisting of nucleotide base multinomial probabilities for each position in the pattern and different probabilities for background positions outside the pattern. In another view, the motif finding problem can be classified as exact motif finding (without insertions and deletions) and inexact motif finding (with insertion and deletions). Karci (2009) proved that exact motif finding is a P-type problem and this can be solved using deterministic method. The inexact motif finding problem can be solved using stochastic or approximate methods.

Among those previous works, most popular being is the Multiple Em for Motif Elicitation (MEME) system (Bailey and Elkan 1994), Gibbs sampler (Thompson et al. 2003) and CONSENSUS (Hertz et al. 1990). Even with weak signals, the methods such as MEME and Gibbs Motif Sampler effectively find motifs of variable width and occurrences in DNA and protein sequences.

Many other algorithms have been developed to improve these popular motif discovery tools by means of performance, length of motifs or some other considerations. Liu et al. employed genetic algorithm for finding potential motifs in the regions of TSS (Liu et al. 2004). Structured genetic algorithm is used to discover highly conserved motifs among upstream sequences of co-regulated genes (Stine et al. 2003). The GA-based hybrid schemes have also been proposed. One such method is GARPS that combines GA and Random Projection Strategy (RPS) to identify planted (l, d)-motifs. In this paper, RPS is used to find good starting positions by introducing position-weighted function, followed by GA that is used to refine the initial population obtained from RPS (Huo et al. 2010).

Recently, algorithms based on promoter sequences of co-regulated genes and phylogenetic footprinting had been suggested. These algorithms integrate two important aspects of a motif’s significance into one probabilistic score. These aspects are overrepresentation of motifs and cross-species conservation of motifs. Wang and Stormo (2003) developed the motif finding algorithm PhyloCon that takes into account both aspects, conservation among orthologous genes and co-regulation of genes within a species. Sinha et al. (2004) developed the algorithm PhyME that was based on a probabilistic approach. This algorithm handles data from promoters of co-regulated genes and orthologous sequences.

## Materials and methods

### Problem statement

According to reference (Chan et al. 2008), the motif identification in unaligned DNA sequences using GAs can be defined as follows:

*Input* A set of *N* sequences *S* = {*S*_1_, *S*_2_,*…*, *S*_*N*_}, each of which is from the finite alphabet *D* = {*A*, *T*, *C*, *G*}, where the length of each sequence is *l*, and the motif width *w* with a constraint 0 < *w* ≪ *l.*

*Output* A set of motifs, where each motif is represented by a set of subsequences *M* = {*m*_1_, *m*_2_,*…*,*m*_*N*_}, and each *m*_*i*_ is a subsequence with length *w* from sequence *S*_*i*_. The set of motifs is such that the consensus similarity score or/and the length of the motif is maximized.

### The method

Genetic algorithm (GA) is a widely used evolutionary algorithm, which applies a stochastic optimization technique. It operates on a population of candidate solutions to a specific problem domain. Specifically, the structure in the current population is evaluated for its effectiveness as a solution during each generation. Based on this evaluation, a new population of candidate structures is formed using operators like crossover and mutation. This process is iterated until an optimal solution is found or no improvement is achieved after a significant amount of evaluations. (Goldberg 1989).

The other genetic algorithms proposed for motif identification like “finding motifs by genetic algorithm (FMGA)” (Liu et al. 2004) is a single objective genetic algorithm that can identify a single motif of fixed length. The structured GA (Stine et al. 2003) that used a tree-structure chromosomal representation in the algorithm can identify the motif of variable length. The proposed algorithm is a multi-objective approach for optimizing a vector-valued cost function. This niched Pareto genetic algorithm is able to identify de novo multiple motifs of variable lengths simultaneously.

The multi-objective optimization seeks to optimize the components of a vector-valued cost function. In single objective optimization, the solution of the problem is a single global optimum point, where as in multi-objective optimization, the solution of the problem is a set of points known as the Pareto-optimal set. Each point in this set is optimal in the sense that no improvement can be achieved in one cost vector component that does not lead to degradation in at least one of the remaining components. According to reference (Fonseca and Fleming 1993), assuming a maximization problem, the following conditions apply:

#### Condition 1 (inferiority)

A vector **u** = (*u*_1_,…,*u*_*n*_) is said to be inferior to **v** = (*v*_1_,…,*v*_*n*_) iff **u** is partially less than **v** (**u***p* < **v**), i.e., 

#### Condition 2 (superiority)

A vector **u** = (*u*_1_,…,*u*_*n*_) is said to be superior to **v** = (*v*_1_,…,*v*_*n*_) iff **v** is inferior to **u**.

#### Condition 3 (non-inferiority)

Vectors **u** = (*u*_1_,…,*u*_*n*_) and **v** = (*v*_1_,…,*v*_*n*_) are said to be non-inferior to one another if **v** is neither inferior nor superior to **u**.

Each element in the Pareto-optimal set constitutes a non-dominant solution to the multi-objective problem. These solutions are non-dominant as there are no other solutions superior in all attributes.

In the context of our algorithm, the vector **u** and **v** are representing the fitness of motifs, which has two elements. The first element is the similarity score of the consensus motif among the given co-regulated promoter sequences, and the second element of the vector is the length of the motif. To compare the fitness of motifs, we compare the motifs element by element.

#### Consensus similarity

A pattern of nucleotides that is represented by maximum frequency at a position is called the consensus string. To measure the similarity score, we used the normalized similarity of a consensus motif.

#### Length of the motif

We used the normalized length as the measure of the size of a motif. The normalized length of a motif is defined as the length of a motif divided by the maximum possible length of motifs.

### The algorithm

**Fig. 1:**
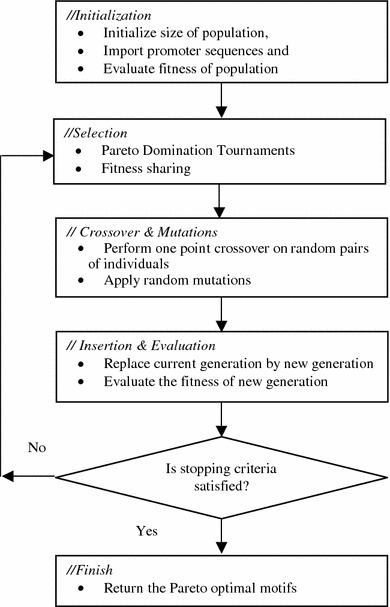
Flowchart of the niched Pareto genetic algorithm

Figure 1 illustrates the working of niched Pareto GA. The key components of the algorithm are initialization, selection, crossovers & mutation, insertion and evaluation and finish. The initialization step deals with the representation of the motifs using a suitable encoding scheme and the initialization of the population. The selection step selects the suitable candidate motifs for the reproduction from the current population. The crossover and mutation step deals with the generation of new offsprings and adaption of the environmental influences. The fitness of newly generated offsprings is evaluated using an objective fitness function, and the fit offsprings are inserted in the population. During each generation of the evolutionary process, each member of the population is evaluated by the objective fitness function. The evolutionary process stops when the stopping criteria are satisfied.

#### Initialization

**Fig. 2:**
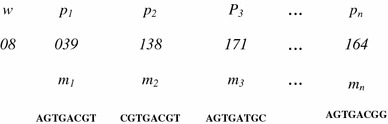
Representation of a member: *p*_*i*_ is the starting position of the subsequence *m*_*i*_ of length *w*, in *i*th sequence

To represent an individual motif, we used the position-based representation approach as used in the algorithms GALF-P and GAME (Chan et al. 2008; Wei and Jensen 2006). Here, each individual motif is represented by a vector *P* = {*w*, *p*_1_, *p*_2_,…,*p*_*N*_} storing the length of motif and a set of possible starting positions for the motif instances in each sequence. Vector P is used to generate the vector of subsequences for a possible consensus solution set *M*, where each subsequence is of length *w.* The consensus solution set *M* = {*m*_1_, *m*_2_,…,*m*_*N*_}, where each *p*_*i*_ is uniquely mapped to subsequence *m*_*i*_ of length *w*, is used to generate the consensus motif. Figure 2 illustrates this approach. The initial population is generated using this multiple attribute representation. The representation of an individual motif in the algorithm is having two fields (1) the length of motif and (2) the starting positions in the promoter sequences. Hence, the population has all the members of same size but having different value of attribute length. This enables the algorithm to identify the motifs of variable length. The numeric encoding is used to represent the width and the starting position of a subsequence. The size of the population is taken from the user as an input. The algorithm randomly generates the initial population of the size specified by the user. The length of motif and starting positions of motif for each subsequence are randomly generated.

#### Selection

Maintaining population diversity and selective pressure is the key issue while using a selection method. The tournament selection is one of the most common methods used in GAs. In this method, two individual motifs are chosen randomly from the current population, and the one with higher fitness score is selected for the reproduction. But this binary tournament selection assumes a single solution of the problem and GA converges to a single global optimum. To obtain Pareto-optimal solutions, we used the selection scheme as proposed by Horn et al. (1994). This scheme uses Pareto domination tournaments for selection and fitness sharing, when there is non-dominant tournament.

##### Pareto domination tournaments

In this scheme, two candidate motifs are chosen for selection at random from the population. A comparison set (of size *t*_dom_) of motifs is also chosen randomly from the population. Each of the candidate motifs is then compared against the motifs of the comparison set, and a non-inferior candidate motif is selected for reproduction. If there is a tie, means neither or both of the candidate motifs are non-inferior, then sharing is used to decide the winner.

##### Fitness sharing

Goldberg and Richardson (1987) introduced the concept of fitness sharing. The aim of fitness sharing is to distribute the population in search space over a number of different peaks, which are possible Pareto-optimal solutions. So, fitness sharing helps the algorithm to maintain the population diversity. Due to this sharing, fitness of an individual motif is derated. The derated fitness of an individual motif is calculated by taking its unshared objective fitness *f*_*i*_ and dividing it by the *niche count*, which is an estimate of the size of the neighborhood of an individual motif *i.* The neighborhood of a motif is computed by counting how many individual motifs in the population have the consensus similarity score and the motif length similar to the motif in consideration. After sharing the fitness of individual motif is *f*_*s*_(*i*) = *f*_*i*_*/q*_*i*_, where *q*_*i*_ is the *niche count*.

#### Crossovers and mutation

To generate new offspring from their parents, we used one-point crossover method. In this method, a crossover point less than the length of motif is randomly generated. Then, after the crossover point, the sub-strings representing the parents are swapped.

There may be chances of being trapped in a local optima and getting the false motif. To avoid this, we used mutation. Mutation also helps in maintaining population diversity and fast convergence of GA. To produce the mutation effect, first we randomly select a victim individual motif, which is going to be mutated and then changed its starting position value.

#### Insertion and evaluation

In the context of genetic algorithm, the fitness of a motif represents how good the individual as an optimal solution is. We use the two objectives to measure the fitness of a consensus motif in the population. The first objective is the similarity score of the consensus motif among the given co-regulated promoter sequences, and the second objective is the length of the consensus motif. Our algorithm tries to search the Pareto-optimal solutions that maximize both objectives.

##### Consensus similarity

**Fig. 3:**
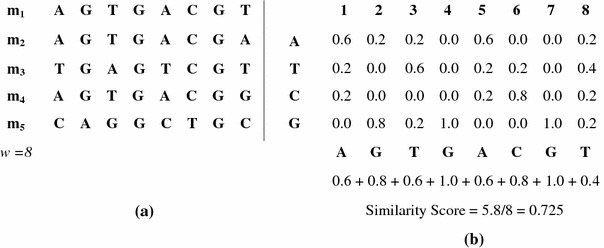
A consensus motif representation and its similarity score computation, **a** the consensus solution set *M*, **b** the consensus motif and similarity score

To measure the similarity score, we used the normalized similarity of a consensus motif that is generated by a member of population. The consensus similarity score is computed using the PWM (position weight matrix) of each individual motif. This is defined as:1where *M* is a consensus motif, *w* is the length of motif, and *f*_max_(*j*) is the maximum frequency value in column *j*. This approach is explained in the Fig. 3.

##### Motif length

A motif of large length is having less probability of appearing in a sequence just by chance. So, a motif of large length is less likely to be a false motif. We used the normalized length as the measure of the size of a motif. We assumed the minimum length of a motif is 4 and maximum length of the motifs is 20.

#### Finish

The algorithm uses two stopping criteria. The first is criterion is stagnation—if there is no or marginal (below threshold) improvement in the average fitness of the population, from one generation to the next, for ten consecutive generations. The second criterion is when the algorithm completes the specified number of generation cycles. On completion, the algorithm returns the Pareto-optimal set of motifs.

## Simulation results

In order to evaluate the performance of our algorithm for motif identification, we used the synthetic data sets comprising various scenarios. Synthetic data sets are made of the following combinations: (1) the number of sequences: 8–20, (2) length of the sequences: 200–500 bp, (3) size of motifs: 4–20, (4) the background distributions: uniform, AT-rich & GC-rich and (5) motif conservation levels: high or low.

The data sets with uniform background distribution have equal probability of occurrences of A, C, G and T. The AT-rich data sets have 60% AT content & 40% GC content, whereas GC-rich data sets have 60% GC content and 40% AT content. We embedded each sequence with the instances of known motifs at random positions. The known motifs that are embedded in the sequences generated randomly by following two conservation levels: high and low. A high conservation motif is formed such that at any position a dominant nucleotide has a probability of 0.91 and each of the rest is 0.03. A low conservation motif is formed such that at any position, a dominant nucleotide has a probability of 0.70 and each of the rest is 0.10. The algorithm is implemented using the Java programming language. Since Java is the platform independent and architecture neutral language, the program can be run on any kind of processor and operating system. The program has a graphical user interface to take the input from the user and to display the output. The program is free and available on request through e-mail to the corresponding author.

To evaluate the algorithm’s ability for the identification of multiple motifs, we embedded some data sets with multiple known motifs of the variable length and conduct a number of runs. The modifiable parameters of the algorithm are the number of promoter sequences, the size of population, the number of generation cycles, the probability of crossover and the probability of mutations. The probability of crossover represents the probability of generation of new offsprings by the members selected for reproduction. The probability of mutation represents the probability of modification of position values in the population. We compared the motifs retrieved by algorithm with original implanted motifs. The motifs instances in the real data sets are not exact. There may be variation because of mutations and deletions. Also there are some other sources of noise like false reported sequence or motifs in databases. The motif instances, we have embedded, are not exact. It means that we have embedded the motif pattern in the promoter sequences that may vary from sequence to sequence. So in the cases where we found motif instances of more than 70% consensus similarity, we considered this as threshold for successful identification. Since a low conservation motif is formed such that at any position, a dominant nucleotide has a probability of 0.70. We have taken 70% consensus similarity as threshold.

For each simulated data set, to evaluate the performance of our algorithm, we used the standard information retrieval parameters, precision (sensitivity) and recall (specificity) (Hu et al. 2005). Precision *P* is number of predicted motif sites that are true sites divided by the number of predicted motif sites, and recall *R* is number of predicted motif sites that are true sites divided by the number of true sites. These two parameters are combined to compute the standard parameter for comparison *F* score, as follows:2

High values of *F* occur only when both precision and recall are high. The average of precision, recall and *F* score were calculated for the discovered motifs for each data set. We also compared the performance of the algorithm with MEME. Results of various scenarios such as the number of sequences, length of sequences, and number of motifs identified, length of motifs, precision, recall and *F* score for each simulation condition are shown in Table 1. The better *F* score has been marked bold. The *F* score for motif identification is up to 0.824 for high conservation of motifs and 0.710 for low conservation of a motif. The algorithm finds motifs of different length with best similarity score for each length. The number of motifs returned by the algorithm is depending upon how many non-inferior motifs are present in the input data sets. The algorithm returns only non-inferior solutions. The algorithm has limitations in identifying multiple motifs of the same length. If there are multiple motifs of same length in the data sets, our algorithm finds only one with best similarity score. Also if there are multiple motifs of same similarity score, the algorithm finds the longest one. Results show that the *F* score is better for long motifs in comparison with short motifs. The *F* score is better for long motifs because these motifs are less likely to be a false motif and having less probability of occurring in the sequences just by chance.

**Table 1:** Results of various scenarios for multiple motifs

S. no.	*N*	*L*	*nM*	C	*w*	MEME			Niched Pareto GA		
						Precision	Recall	*F* score	Precision	Recall	*F* score
1	08	200	01	H	08	0.875	0.875	0.875	0.750	0.750	0.750
2	08	200	02	H	06	0.667	0.889	0.762	0.636	0.778	0.700
				L	09	0.538	0.778	0.636	0.583	0.778	0.667
3	12	300	01	L	10	0.600	0.750	0.667	0.643	0.750	0.692
4	12	300	02	H	07	0.750	0.750	0.750	0.667	0.667	0.667
				L	11	0.625	0.625	0.625	0.733	0.688	0.710
5	14	300	02	L	07	0.533	0.571	0.552	0.643	0.643	0.643
				H	11	0.688	0.786	0.733	0.786	0.786	0.786
6	14	400	03	L	07	0.529	0.643	0.581	0.600	0.643	0.621
				H	12	0.688	0.786	0.733	0.786	0.786	0.786
				H	09	0.611	0.733	0.667	0.733	0.733	0.733
7	16	400	02	L	14	0.579	0.688	0.629	0.667	0.750	0.706
				H	14	0.813	0.813	0.813	0.750	0.750	0.750
8	16	500	03	H	06	0.667	0.750	0.706	0.688	0.688	0.688
				L	12	0.600	0.667	0.632	0.706	0.667	0.686
				H	15	0.667	0.750	0.706	0.778	0.875	0.824

*N* number of sequences, *L* length of sequences, *nM* number of motifs embedded, *C* conservation of motif, *w* length of embedded motifs, *H* high, *L* low

We also tested this algorithm with the real biological data sets. We used the promoter sequence data of *Saccharomyces cerevisiae*. We run this algorithm against ten target genes of transcription factor MIG1, nine target genes of transcription factor GCN4, seven target genes of PDR3 transcription factor and six genes of MCB transcription factor. The experimentally reported consensus motifs and motifs identified by niched Pareto GA algorithms are shown in Table 2. Here, we have shown the motif that matches best with experimentally reported motifs. The results show that the algorithm can effectively identify multiple motifs if present in the sequences.

**Table 2:** Results of biological promoter sequences

S. no.	TF data set	Reported consensus motif	Discovered motif by niched Pareto GA
1	MIG1	TTATTTCTGGGGTA	TTATTGCTGGGGTA
		CCCCAGATTTT	CTCCAGATTTTC
2	GCN4	TGACTCA	ATGACTCTT
			TGAGTC
3	PDR3	TCCGCGGA	TTCCGCGGAA
4	MCB	ACGCGT	ACGCGT

## Conclusion

Identification of transcription factor binding sites is an important and difficult problem. Most of the existing methods such as Gibbs sampling algorithm are local search methods, so they may suffer from the problem of local optima. Genetic algorithm provides a good approach to solve this problem. Genetic algorithm solves the optimal problem based on the biological characteristics. In this paper, we have used the multi-objective genetic algorithm that produces Pareto-optimal solution set in place of a single optimum solution.

Simulation results of the algorithm on synthetic data comprising various scenarios show that the algorithm is able to predict the motifs with average *F* score in the range of 0.621–0.824. The algorithm is also able to detect multiple motifs of variable length present in the sequences. The results show that the algorithm can identify motifs in the promoter data of *S. cerevisiae* effectively.

The performance of this approach can probably be improved using more intelligent operators for selection, crossover and mutation. Currently, the algorithm can find multiple motifs of variable length, but in the case of multiple motifs of the same length, it finds the single motif with maximum consensus similarity score. However, this issue can be addressed using a ranking scheme of solutions. On the other hand, the fitness evaluation can be improved if we are able to additionally incorporate terms that reflect the biological messages behind the similarities among motifs.
